# The importance of vitamin D levels in patients with inflammatory bowel disease

**DOI:** 10.1007/s13105-025-01096-5

**Published:** 2025-05-26

**Authors:** Evgenia Koureta, Pantelis Karatzas, Panagiotis Kanellopoulos, Angeliki Papapanagiotou, Vasileios Lekakis, Giorgos Bamias, Andreas Koutsoumpas, George Karamanolis, Jiannis Vlachogiannakos, Athanasios G. Papavassiliou, George V. Papatheodoridis

**Affiliations:** 1 1st Department of Gastroenterology, Medical School, National and Kapodistrian University of Athens, General Hospital of Athens “Laiko”, Athens, Greece; 2 Department of Biological Chemistry, Medical School, National and Kapodistrian University of Athens, General Hospital of Athens “Laiko”, Athens, Greece; 3 GastreonteroIogy Unit, 3rd Department of Internal Medicine, Medical School, National and Kapodistrian University of Athens, General Hospital of Athens “Sotiria”, Athens, Greece

**Keywords:** Vitamin D, Cholecalciferol, Crohn’s disease, Ulcerative colitis, Inflammatory bowel disease

## Abstract

The possible role of vitamin D (VD) in the pathogenesis of inflammatory bowel disease (IBD) and the associations between VD levels and IBD activity remain unclarified. We aimed to assess VD levels in IBD patients and their associations with IBD activity. We evaluated VD levels in Greek patients aged 18–75 years old with Crohn’s disease (CD) or ulcerative colitis (UC). Patients were ineligible under the following conditions: history of enterectomy/right colectomy, receiving VD or agent(s) interfering with VD metabolism during the last three months and any comorbidities that influence VD levels. Epidemiologic characteristics, clinical course, laboratory investigations, endoscopic and histologic findings were recorded. In total, 122 patients with CD and 71 with UC were included. Most of them had low levels of VD (90% of CD and 91.5% of UC patients). Patients with clinically active CD or UC had lower levels of VD compared to those in remission (*p* = 0.009 and *p* = 0.033, respectively).CD patients with low levels of VD had higher CRP and stool calprotectin compared to those with normal levels of VD (P = 0.032 and *P* = 0.002, respectively). In UC, patients with pancolitis had lower VD levels compared to patients with proctitis (*P* = 0.036). In conclusion, the majority of Greek IBD patients have low levels of VD. Clinical activity is related to lower levels of VD. Low compared to normal levels of VD in CD patients are associated with higher CRP and calprotectin levels, so VD levels might serve as an activity marker.

## Introduction

The etiopathogenesis of inflammatory bowel diseases (IBD), Crohn's disease (CD) and ulcerative colitis (UC), remains unclear, but they are currently considered as autoimmune disorders developing in susceptible hosts with a specific genetic background as response to intestinal environmental factors including microbes [35]. Environmental factors seem to be associated with IBD development, as the prevalence and incidence differ between northern and southern countries as well as between western and eastern countries [20, 32]. Moreover, there is evidence that migration from areas with low prevalence to regions with higher IBD prevalence is associated with a corresponding increase in disease cases [34].

VD is a fat-soluble vitamin absorbed from the small intestine, particularly from the jejunum. Beyond its role as a regulator in the metabolism of calcium and phosphorus, it also plays a significant role as a regulator of innate and adaptive immunity [23]. VD is considered to suppress the pro-inflammatory cytokines secreted by T-helper (Th)1 lymphocytes, such as interferon (IFN)-γ, interleukin (IL)−2 and tumor necrosis factor (TNF)-α which play a crucial role in the pathogenesis of IBD. On the other hand, VD may support the development of Th2 lymphocytes, which secrete anti-inflammatory cytokines such as IL-4, IL-5, and IL-13. VD also inhibits the development and production of pro-inflammatory IL-17 by Th17 lymphocytes [14, 23].

This immunomodulatory role of VD and especially its potential role in IBD pathogenesis has attracted research interest. However, it is not clear whether the reduction in VD precedes and is etiopathogenetically related to the development of IBD or whether it is a consequence of inflammation and inadequate absorption from the intestinal lumen [18]. There are conflicting reports in the literature about the association of VD levels with clinical parameters of IBD, as well as with the effect of biologic therapies, specifically anti-TNF agents [6, 29, 33]. Therefore, the aim of this study was to assess VD levels in IBD patients and their associations with markers of IBD activity.

## Material and methods

We included 122 patients with CD and 71 patients with UC aged 18–75 years, who had a regular follow-up at our tertiary IBD centre during 2014–2020. Patients were excluded from the trial if they had a history of right colectomy or enterectomy, if they were receiving VD or agents interfering with VD metabolism during the last three months and if they had any comorbidities that could influence VD levels. We also included 44 healthy individuals who constituted the control group; they visited our outpatient gastroenterology clinics in order to plan a screening colonoscopy or suffering from functional disorders. There was no difference between patients and controls regarding mean age and gender distribution. This study was performed in line with the principles of the Declaration of Helsinki. Approval was granted by the Ethics Committee of the General Hospital of Athens “Laiko” and of Medical School of National and Kapodistrian University of Athens.

We recorded epidemiologic characteristics of patients including age, sex, height, weight, smoking status and Montreal classification of the disease. Additionally, we recorded flares, hospitalizations due to the disease and surgeries related to IBD up to one year after the date of the sample collection. We also analyzed laboratory investigations on the day of the sample collection including stool calprotectin, C-reactive protein (CRP), white blood count, hematocrit, platelets, erythrocyte sedimentation rate (ESR), albumin and ferritin. Current and previous treatments during the course of the disease, endoscopic and histologic reports were also recorded. For all patients, we had available clinical indexes of activity: Harvey-Bradshaw Index (HBI) for CD and Simple Clinical Colitis Activity Index (SCCAI) for UC and/or clinical Mayo score when endoscopy was performed on the day of sample collection.

In CD, clinical remission was defined as HBI < 5 and endoscopic remission as absence of mucosal ulceration. Absence of inflammation, intraepithelial neutrophils, erosion, ulceration, or epithelial damage was used to define histological remission. In UC, Mayo score ≤ 1 or SCCAI < 1 determined clinical remission and endoscopic Mayo score ≤ 1 defined endoscopic remission. Absence of inflammation, neutrophils, erosions or ulcerations defined histological remission in UC patients.

All the serum samples were centrifuged and stored at −80 °C. Levels of VD in its 25-hydroxy form were determined, as it is considered to represent the best marker of VD status in the human body. VD levels were measured in ng/ml by electrochemiluminescence in Roche (Cobas 801) analyzer. Levels of VD ≥ 30 ng/ml were defined as normal, 21–29 ng/ml as insufficient and ≤ 20 ng/ml as deficient, according to the manufacturer of the reagents.

### Statistical analysis

Statistical analyses were performed with SPSS package (IBM® SPSS® Statistics 29.0, SPSS Inc, IBM, Chicago, IL, USA). Descriptive statistics for categorical variables are shown as frequencies and percentages. The mean value ± standard deviation (SD) was given for continuous variables with normal distribution, whereas skewed continuous variables were expressed by median values with interquartile ranges (IQR). Categorical comparisons were carried out using either the corrected chi-squared test or two-sided Fisher’s exact test. Differences between groups for normally distributed values were examined using the Student’s t-test or one-way ANOVA. The Mann–Whitney U test or Kruskal–Wallis test was applied for non-normally distributed values. Correlations between quantitative variables were evaluated using Pearson or Spearman correlation coefficients. All tests were two-tailed and P values < 0.05 were considered to be statistically significant.

## Results

### VD in patients with CD

The main characteristics of all patients with CD as well as in relation to low and normal VD levels are provided in Table 1, whereas the mean VD levels in relation to the characteristics of patients with CD are shown in Table 2. There was no statistically significant difference of VD levels between patients with CD and the controls (18.7 ± 7.5 vs 20 ± 8 ng/ml, *P* = 0.461).

**Table 1:** Main characteristics of patients with Crohn’s disease (CD) in relation to vitamin D (VD) levels

	Total (*N* = 122)	Low VD levels (*n* = 110)	Normal VD levels (*n* = 12)	*P*
Age, years	37.7 ± 15.9	37.5 ± 15.4	40.3 ± 20.9	0.075
Age at CD diagnosis, years	31.2 ± 15.9	31.1 ± 16.2	31.9 ± 21.2	0.276
Females, *n* (%)	44 (36)	42 (38.2)	2 (16.7)	0.208
Body mass index, kg/m^2^	24.8 ± 4.9	24.8 ± 4.9	25.0 ± 3.4	0.332
Smokers, *n* (%)	34 (28)	30 (27.3)	4 (33.3)	0.737
Disease duration, years	6.6 (7.9)	6.4 (7.8)	8.4 (9.1)	0.325
Harvey-Bradshaw Index	2.1 ± 1.8	2.2 ± 2.3	1.5 ± 1.4	0.306
C-Reactive Protein, mg/l	7.1 (10.1)	7.4 (10.5)	3.4 (2.1)	0.032
Stool calprotectin, μg/mg	180 (539)	209 (582)	75 (105)	0.002
Location, *n* (%)—L1 L2 L3 L4	42 (34.4) 20 (16.4) 31 (25.4) 29 (23.7)	35 (31.8) 19 (17.3) 27 (24.5) 29 (26.3)	7 (58.3) 1 (8.3) 4 (33.3) 0	0.183
Behaviour, *n* (%)—B1 B2 B3	70 (57.4) 43 (35.2) 9 (7.4)	65 (59) 38 (34.5) 7 (6.4)	5 (41.7) 5 (41.7) 2 (16.7)	0.319
Perianal disease, *n* (%)	27 (22)	23 (20.9)	4 (33.3)	0.462
Endoscopically active, *n* (%)	63 (51.6)	57 (51.8)	6 (50)	1.000
Histologically active, *n* (%)	61 (50)	57 (51.8)	4 (33.3)	0.566
Treatment*, *n* (%) Biologic agent(s) ± other agents AZA without biologic agent Steroids ± mesalazine Mesalazine only	72 (71.3) 11 (10.9) 8 (7.9) 10 (9.9)	67 (71.3) 10 (10.6) 8 (8.5) 9 (9.6)	5 (71.4) 1 (14.3) 0 1 (14.3)	0.844

Quantitative variables are expressed as mean ± SD or median (IQR) values

^*^Twenty-one patients were under no therapy

**Table 2:** Mean ± SD vitamin D (VD) levels in relation to main characteristics of patients with Crohn’s disease (CD)

Patient characteristics	VD levels (ng/ml)	*P*
Age, < 40 years (*n* = 89) ≥ 40 years (*n* = 33)	19.7 ± 6.9 16.1 ± 8.3	0.017
Gender, males (*n* = 78) females (*n* = 44)	19.3 ± 8.1 17.6 ± 6.3	0.215
Body mass index, ≤ 25 kg/m^2^ (*n* = 67) > 25 kg/m^2^ (*n* = 55)	19.4 ± 6.7 17.9 ± 8.3	0.280
Smoking, yes (*n* = 34) no (*n* = 88)	16.9 ± 8.5 19.4 ± 6.9	0.095
Disease duration, ≤ 3 years (*n* = 58) > 3 years (*n* = 64)	18.3 ± 7.9 19.1 ± 7.1	0.598
Harvey-Bradshaw Index, < 5 (*n* = 108) ≥ 5 (*n* = 14)	19.4 ± 7.5 13.9 ± 5.4	0.009
C-Reactive Protein, ≤ 5 mg/l (*n* = 83) > 5 mg/l (*n* = 39)	19.9 ± 7.6 16.2 ± 6.8	0.011
Stool calprotectin, ≤ 180 μg/mg (*n* = 47) > 180 μg/mg (*n* = 41)	20.4 ± 7.9 17.1 ± 6.7	0.038
Location, L1 (*n* = 54) L2 (*n* = 20) L3 (*n* = 48)	18.5 ± 8.2 18.1 ± 7.7 19.1 ± 6.6	0.855
L4, yes (*n* = 29) no (*n* = 93)	18.3 ± 6.1 18.9 ± 7.9	0.751
Behaviour, B1 (*n* = 70) B2 (*n* = 43) B3 (*n* = 9)	18.5 ± 7.2 18.2 ± 7.7 23.4 ± 7.7	0.146
Perianal disease, yes (*n* = 27) no (*n* = 95)	20.5 ± 8 18.2 ± 7.3	0.157
Endoscopic activity, yes (*n* = 63) no (*n* = 27)	17.9 ± 7.3 18.3 ± 6.9	0.788
Histologic activity, yes (*n*– = 61) nο (*n* = 25)	17.2 ± 6.9 18.1 ± 6.8	0.599
Treatment, *n* (%) Biologic agent ± other agents (*n* = 72) Azathioprine without biologic (*n* = 11) Steroids ± mesalazine (*n* = 8) Mesalazine only (*n* = 10)	18.7 ± 7.3 18.3 ± 8.3 17.8 ± 4.1 16.8 ± 7.8	0.878

Low (ie deficient or insufficient) levels of VD were detected in 110 (90%) CD patients. In particular, 76 (62%) patients had deficient and 34 (28%) had insufficient VD levels. CD patients with low compared to those with normal VD levels had significantly higher median CRP (7.4 vs 3.4 mg/l, *P* = 0.032) and calprotectin levels (209 vs 75 μg/mg, *P* = 0.002) as well as a trend for lower mean age (37.5 vs 40.3 years, *P* = 0.075), but they did not differ in any other epidemiological or disease characteristic.

**Fig. 1:**
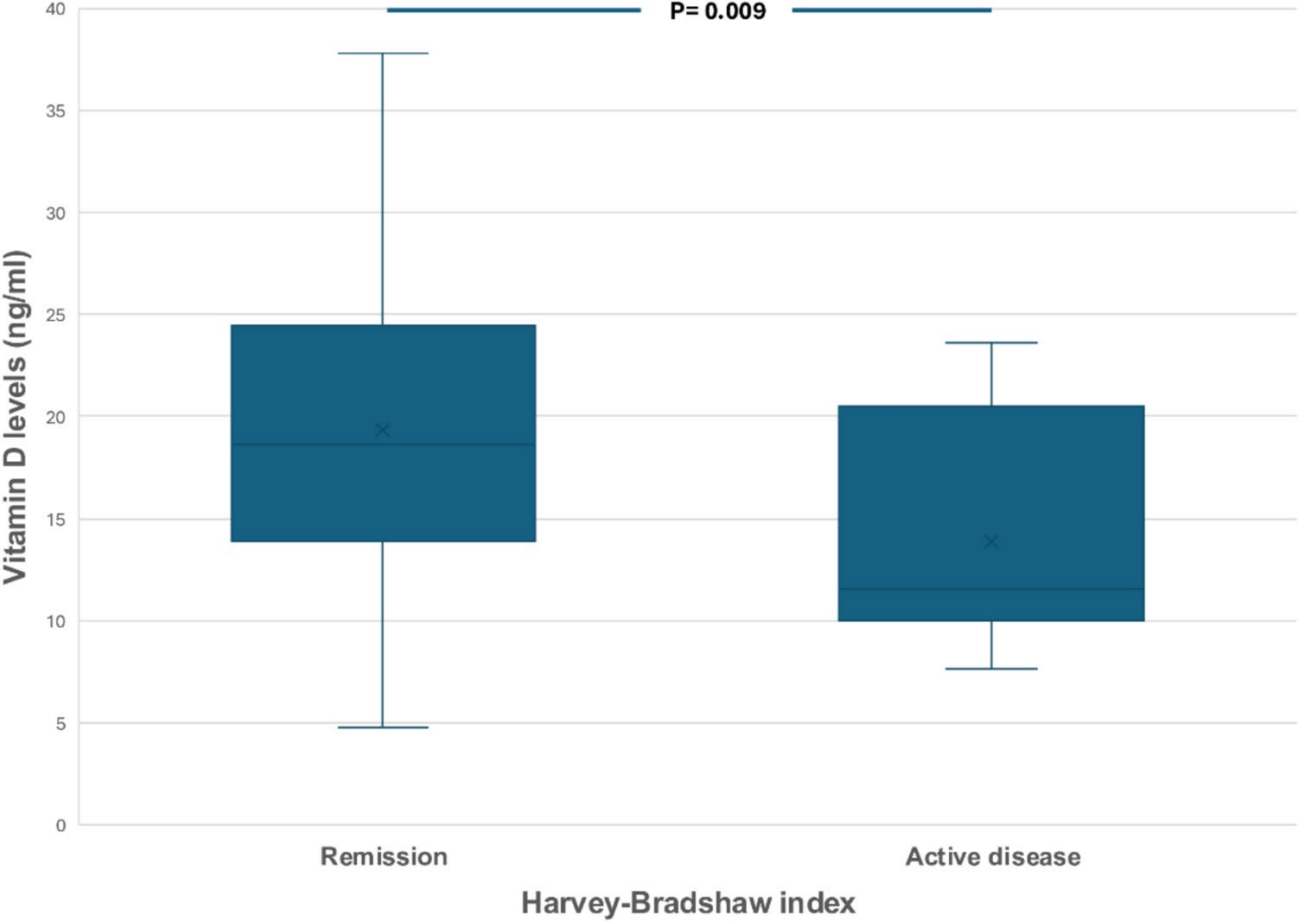
Serum vitamin D levels in Crohn’s disease in clinically active patients or patients in remission according to Harvey-Bradshaw index

Mean VD levels were significantly lower in patients with clinically active CD compared to those in remission (13.9 ± 5.4 vs 19.4 ± 7.5 ng/ml, *P* = 0.009) (Fig. 1) or controls (*P* = 0.023). Moreover, mean VD levels were higher in patients with low than high Harvey-Bradshaw Index (< 5 vs ≥ 5: 19.4 vs 13.9 ng/ml, *P* = 0.009), CRP (≤ 5 vs > 5 mg/l: 19.9 vs 16.2 ng/ml, *P* = 0.011) and stool calprotectin levels (≤ 180 vs > 180 μg/mg: 20.4 vs 17.1 ng/ml, *P* = 0.038). There was no significant difference between VD levels in patients with endoscopic or histologic activity compared to patients with endoscopic or histologic remission. Additionally, there was no difference in VD levels according to body mass index, location or the behavior of the disease based on Montreal classification or the disease duration. Of the remaining characteristics, VD levels tended to be higher in non-smokers compared to active smokers (19.4 vs 16.9 ng/ml, *P* = 0.095).

**Fig. 2:**
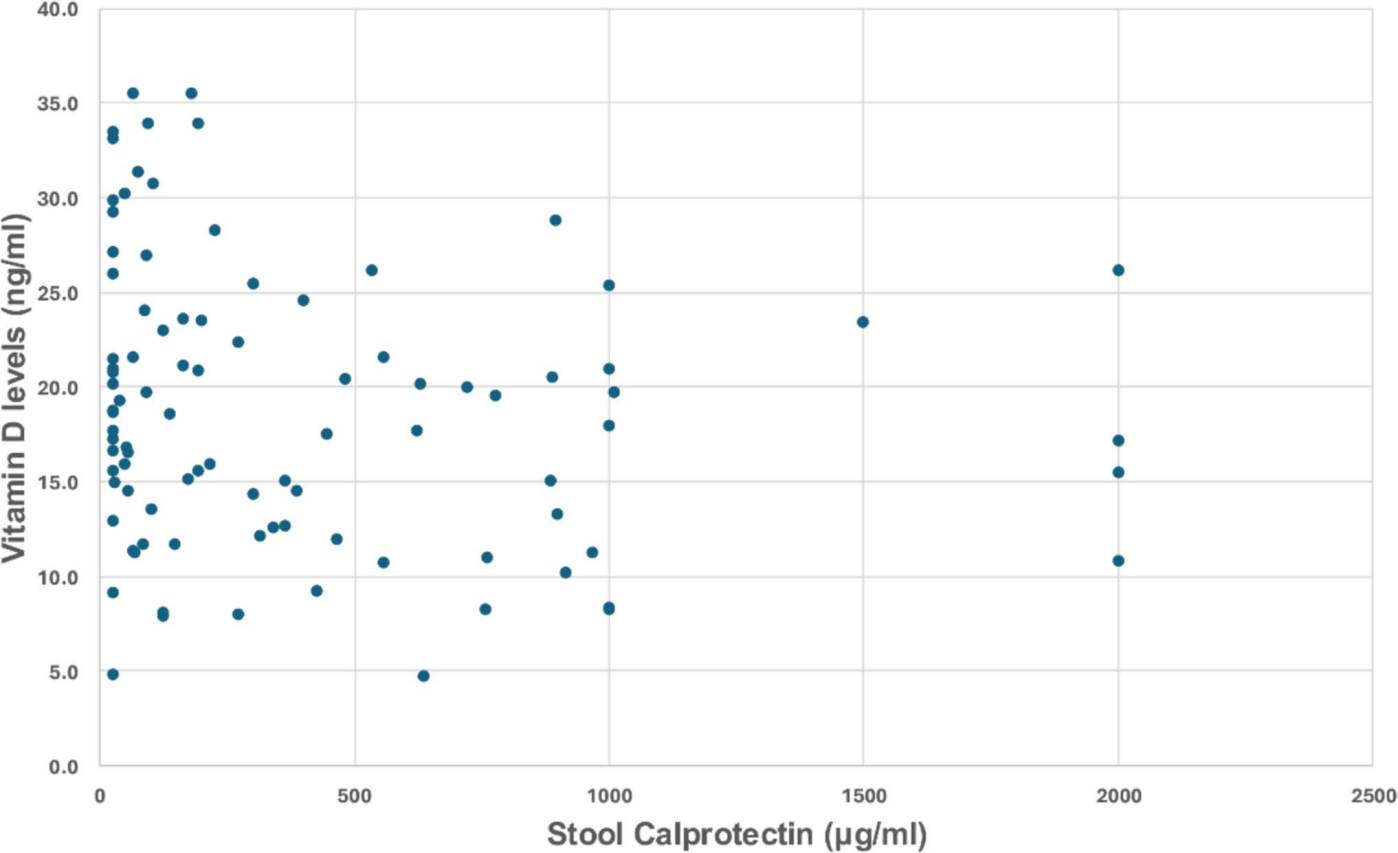
Serum vitamin D levels in Crohn’s disease in correlation with stool calprotectin levels

**Fig. 3:**
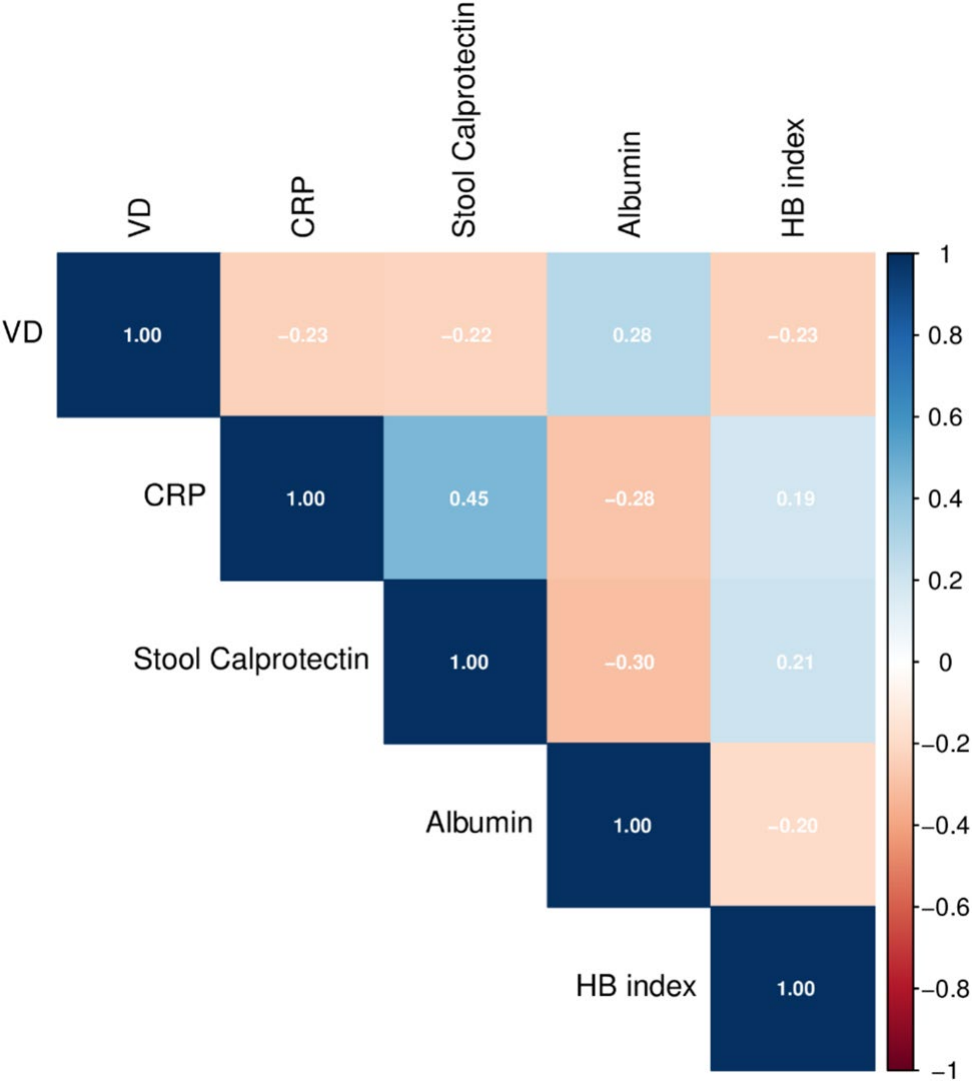
Heat map illustrating the pairwise correlations between various laboratory and clinical characteristics of Crohn’s disease and vitamin D levels

VD levels were significantly correlated with stool calprotectin levels (*r* = −0.22, *P* = 0.042; Fig. 2). VD levels were not correlated with the flares of CD (*P* = 0.219) or number of hospitalizations during the last year (*P* = 0.450). Of the laboratory characteristics, only albumin levels were found to have a positive correlation of VD levels (*r* = 0.284, *P* = 0.002). Concerning extraintestinal manifestations, CD patients with arthropathies had lower mean VD levels compared to patients without (17.2 ± 6.7 vs 19.9 ± 7.9 ng/ml, *P* = 0.046). VD levels from samples taken in autumn or winter were not significantly different from those from samples taken in summer or spring (*P* = 0.405). The pairwise correlations between selected disease characteristics and VD levels are shown in Fig. 3.

### VD in UC patients

The main characteristics of all patients with UC as well as in relation to normal and low VD levels are provided in Table 3, whereas the mean serum levels of VD in relation to the characteristics of patients with UC are shown in Table 4. Similarly with CD patients, there was no difference in the levels of VD between patients with UC and controls (19,3 ± 6.8 vs 20 ± 8 ng/ml, *P* = 0.977), whereas there was also no difference in the levels of VD between UC and CD patients (*P* = 0.364).

**Table 3:** Main characteristics of patients with ulcerative colitis (UC) in relation to vitamin D (VD) levels

	Total (*N* = 71)	Low VD levels (*n* = 65)	Normal VD levels (*n* = 6)	*P*
Age, years	46.7 ± 16.1	47.1 ± 16.3	43.5 ± 13.9	0.612
Age at diagnosis, years	37.3 ± 15.7	37.2 ± 16.0	38.5 ± 13.0	0.846
Females, *n* (%)	25 (35.2)	25 (38.5)	0	0.084
Body mass index, kg/m^2^	25.5 ± 4.4	25.4 ± 4.5	26.4 ± 2.7	0.597
Smokers, *n* (%)	14 (19.7)	13 (20)	1 (16.7)	1.000
Disease duration, years	6 (12)	7 (13.5)	3 (8.5)	0.237
Montreal classification, *n* (%) -E1 - E2 - E3	7 (10) 27 (38) 37 (52)	6 (9.2) 24 (36.9) 35 (53.8)	1 (16.7) 3 (50) 2 (33.3)	0.607
SCCAI	2 (4)	2 (5)	1.5 (3)	0.136
Clinical Mayo score	3 (5)	3 (6)	3.5 (5)	0.681
Endoscopic Mayo score	1 (1)	1 (1)	1.5 (2)	0.463
Endoscopically active, *n* (%)	30 (42.2)	27 (41.5)	3 (50)	1.000
Histologically active, *n* (%)	45 (63.3)	41 (63)	4 (66.6)	0.648
C-Reactive Protein, mg/l	2.5 (3.5)	2.5 (4)	2.5 (1.4)	0.298
Stool calprotectin, μg/mg	87 (437)	88 (455)	25 (251)	0.327
Treatment*, *n* (%) Biologic agent(s) ± other agents AZA without biologic agent Steroids ± mesalazine Mesalazine only	26 (37) 5 (7) 13 (18) 26 (37)	23 (35.3) 5 (7.6) 13 (20) 23 (35.3)	3 (50) 0 0 3 (50)	0.518

Quantitative variables are expressed as mean ± SD or median (IQR) values

SCCAI: Simple Clinical Colitis Activity index, AZA, azathioprine

^*^One patient was under no therapy

**Table 4:** Mean serum levels of vitamin D (VD) in relation to main characteristics of patients with ulcerative colitis (UC)

Patient characteristics	VD levels (ng/ml)	*P*
Age, < 50 years (*n* = 30) ≥ 50 years (*n* = 41)	18.6 ± 7.8 19.8 ± 5.9	0.455
Gender, males (*n* = 46) females (*n* = 25)	20.1 ± 7.3 17.9 ± 5.4	0.182
Body mass index, ≤ 25 kg/m^2^ (*n* = 42) > 25 kg/m^2^ (*n* = 29)	18.5 ± 7.3 20.6 ± 5.7	0.201
Smoking, yes (*n* = 14) no (*n* = 57)	20.8 ± 5.1 19 ± 7.1	0.384
Disease duration, ≤ 6 years (*n* = 36) > 6 years (*n* = 35)	20.3 ± 6.8 18.4 ± 6.7	0.251
Montreal classification, E1 (*n* = 7) E2 (*n* = 27) E3 (*n* = 37)	24.1 ± 5.1 21.0 ± 6.9 17.2 ± 6.3	0.011
Simple Clinical Colitis Activity index, < 1 (*n* = 14) ≥ 1 (*n* = 57)	18.7 ± 5.9 19.5 ± 6.9	0.710
Clinical Mayo score, ≤ 1 (*n* = 9) > 1 (*n* = 52)	21.8 ± 5.6 18.9 ± 6.9	0.033
Endoscopic Mayo score, ≤ 1 (*n* = 31) > 1 (*n* = 30)	19.9 ± 6.2 19.5 ± 7.9	0.823
C-Reactive Protein, ≤ 5 mg/l (*n* = 48) > 5 mg/l (*n* = 23)	19.9 ± 6.8 18 ± 5.8	0.257
Stool calprotectin, ≤ 87 μg/mg (*n* = 23) > 87 μg/mg (*n* = 21)	20.6 ± 7.9 20.2 ± 6.6	0.868
Treatment, *n* (%) Biologic agent(s) ± other agents (*n* = 26) Azathioprine without biologic (*n* = 5) Steroids ± mesalazine (*n* = 13) Mesalazine only (*n* = 26)	17.8 ± 6.2 16.2 ± 7.9 20.1 ± 7 20.9 ± 7	0.283

Overall, 65 (91.5%) UC patients had low levels of VD. In particular, 41 (57.7%) patients had deficient and 24 (33.8%) had insufficient VD levels. UC patients with low compared to those with normal VD levels did not differ in any epidemiological or disease characteristic.

**Fig. 4:**
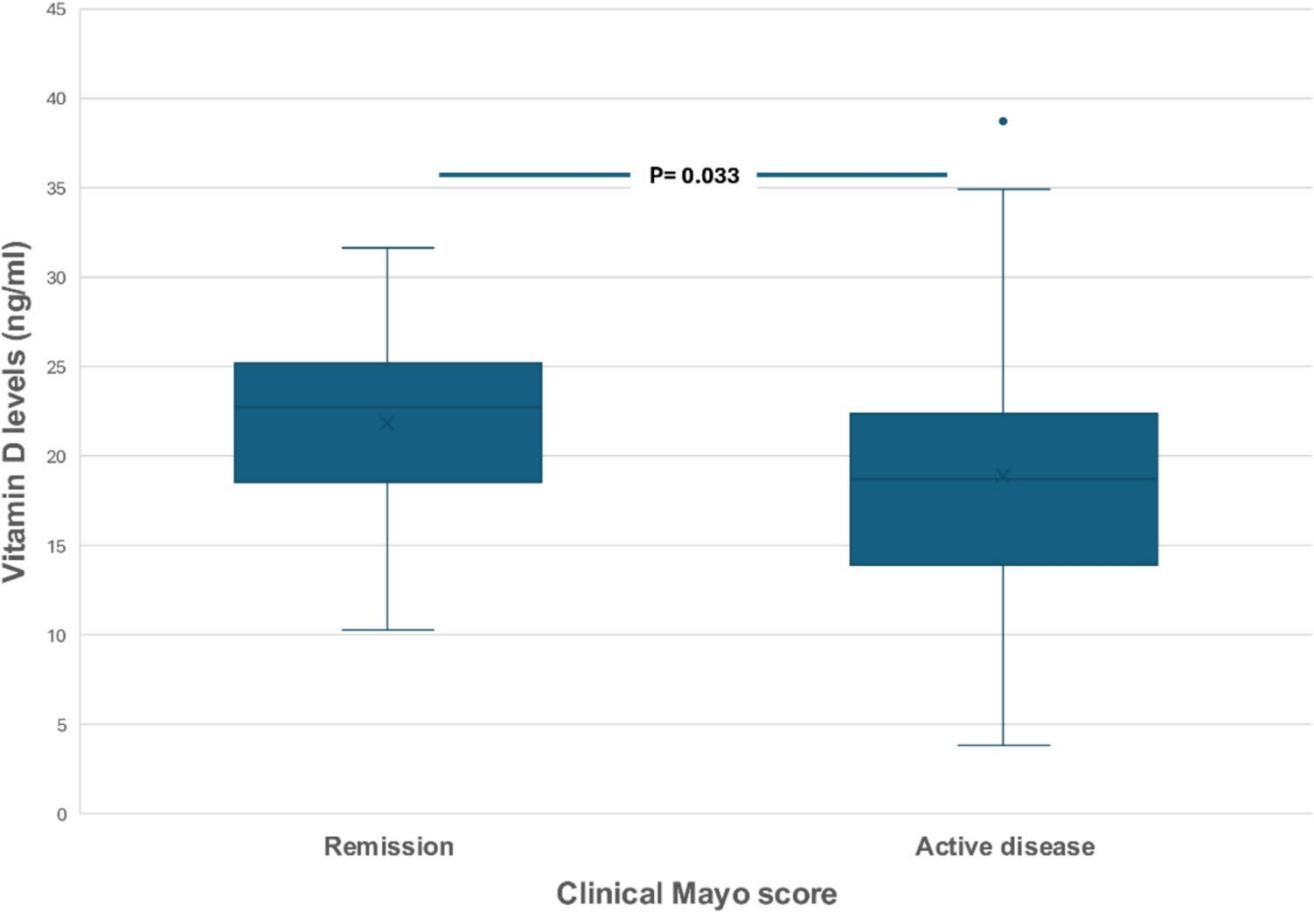
Serum vitamin D levels in ulcerative colitis in clinically active patients or patients in remission according to clinical Mayo score

**Fig. 5:**
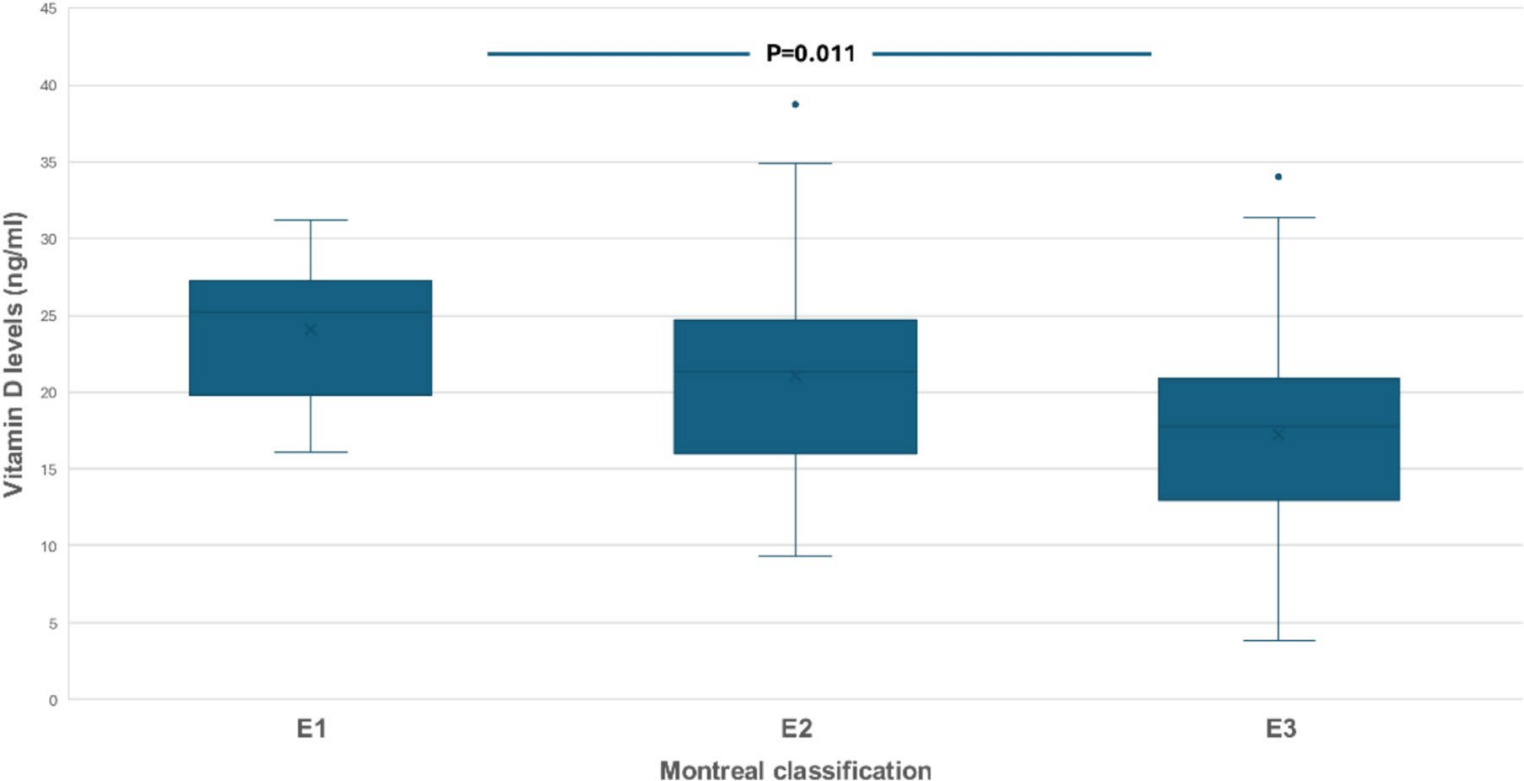
Serum vitamin D levels in ulcerative colitis in relation to Montreal classification

Mean VD levels were significantly lower in UC patients with clinically active disease (according to Mayo score) compared to those in clinical remission (18.9 vs 21.8 ng/ml, *P* = 0.033) (Fig. 4), although there was no difference in VD levels between patients with endoscopically or histologically active compared to those with endoscopic or histological remission. Interestingly, patients with pancolitis had lower VD levels compared to patients with left colitis or proctitis (17.2 vs 21.0 or 24.1 ng/ml, *P* = 0.011) (Fig. 5).

**Fig. 6:**
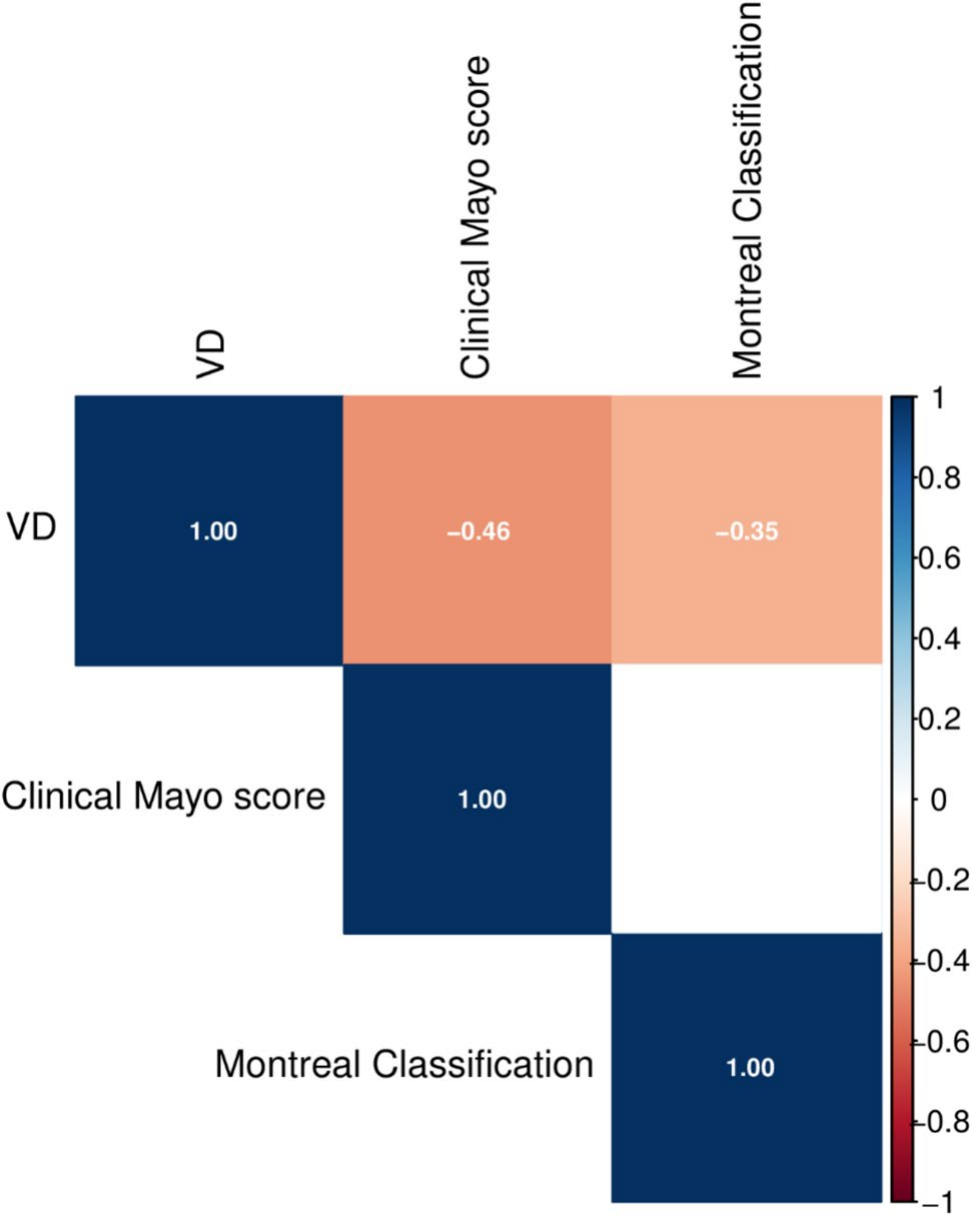
Heat map illustrating the pairwise correlations between clinical features of ulcerative colitis and vitamin D levels

As in patients with CD, no correlation was found between levels of VD in UC patients and flares (*P* = 0.752) or hospitalizations during the last year (*P* = 0.302) or surgery related to UC (*P* = 0.230). In 16 patients who had available serum samples during their treatment with anti-tumor necrosis factor (anti-TNF) agent, VD levels were found to tend to increase under anti-TNF treatment (*P* = 0.070) and to have a trend for positive correlation with the duration of anti-TNF treatment (*r* = 0.163, *P* = 0.074). VD levels from samples taken in winter or autumn were not significantly different from those from samples taken in summer or spring (*P* = 0.526). The pairwise correlations between VD levels and selected clinical features are shown in Fig. 6.

## Discussion

VD is a fat-soluble vitamin and its active form results from the hydroxylation of vitamin D3 or cholecalciferol in two stages: after skin exposure to ultraviolet B radiation from sunlight or after consuming specific foods, especially dairy products and fatty fish. Its metabolism involves not only the liver and kidneys but other tissues including immune system cells as well [17]. In recent years, the consideration of VD as an immunomodulatory factor has gained interest. Given that immune system disturbances are part of the pathogenic mechanisms in IBD, there is growing scientific evidence supporting beneficial effects of its administration related to immunomodulation apart from its established positive effect on bones [23].

Most relevant studies have reported that low VD levels are observed in the majority of patients with IBD, which was also a finding in our study, as > 90% of our patients had low VD levels. Although serum 25(OH)D is the accepted biomarker of VD status in human body, optimal levels are still debated. Most guidelines are based on data concerning the effects of VD in bone metabolism while observational studies indicate levels of 25(OH)D of 20–30 ng/mL to be necessary for the extra-skeletal pleiotropic effects of VD. Especially levels > 30 ng/mL have been suggested to be required for the immunoregulatory function of VD [3]. The differences in proposed optimal 25(OH)D levels are due to several factors. Serum levels of 25(OH)D could be different in the context of testing different populations with various risk factors for deficits. Variations of the clinical patient profiles and the outcomes among studies as well as the absence of standardized laboratory assays contribute to the limitations regarding VD assessments. Thus, the heterogeneity in the cut-off values of 25(OH)D levels in published studies makes results hardly comparable and poses challenges in drawing definite conclusions regarding its role in IBD [27].

Factors leading to low VD levels in IBD patients include reduced sun exposure (e.g. for patients taking thiopurines), reduced activity due to the disease, decreased dairy intake due to intolerance, increased metabolic needs and increased fecal loss. Especially in CD patients, VD malabsorption can result from inflammation in the small intestine especially in terminal ileum. Moreover, patients with severe damage or resection of the terminal ileum exhibit malabsorption of fat-soluble vitamins due to reduced bile acids. IBD patients are also more likely to have reduced bone density. This may be due to both the use of corticosteroids and the low levels of VD, which is traditionally involved in calcium and phosphorus metabolism [9]. It should be noted, however, that low VD levels may be often observed in the general population [31] as well and that our healthy controls had similarly low VD levels with our CD or UC patients.

In most relevant published studies, low levels of VD have been associated with IBD activity [30], which was also observed in our study, and VD supplementation has been suggested to have beneficial effect on disease activity [27].IBD flares have been reported to occur more frequently during winter months potentially associated with lower VD levels, as it has often been reported to occur in autumn/winter compared to spring/summer [13, 21]. Moreover, in northern countries with less sun exposure and thus reduced VD synthesis, the incidence of IBD is higher [20]. On the other hand, we observed no difference in VD levels between samples drawn in winter/autumn and samples drawn in spring/summer, which may be due to the almost all year sunshine in a sunny country like Greece keeping in mind that only 7–30 min of sun exposure are needed for competent VD synthesis [28]. Similarly to our findings, no seasonal variation of VD levels has been reported in a few studies [3, 22], while VD levels were found to be more deficient in summer compared to winter months in another study [24].

Ambiguous are also the data concerning the association of VD levels with flares, hospitalizations and surgeries. In line with the results of our study, Ghaly et al. [12] and Ulitsky et al. [24] reported no association between 25(OH)D3 levels and flares of the disease. In another study [10], CD patients with VD deficiency had more relapses during the previous year compared to those with higher levels. Kabbani et al. [13] reported more visits at the emergency department, hospital admissions, and surgery in patients with low mean VD compared to those with normal mean VD levels.

Data on VD levels and extension of disease are also controversial. In CD, VD levels were reported to be lower in patients with than without small bowel disease [19] or in patients with than without ileum involvement [8]. However, most of the published studies are in line with the results of our study reporting no association between VD levels and location of CD [10, 13, 24] or its behavior [10, 24]. In UC, VD levels were reported to be lower in patients with pancolitis compared to those with smaller extent of the disease [15], which is in agreement with our results. On the contrary, two other studies [10, 13] reported no correlation of VD levels with the extent of UC.

Low VD levels were associated with elevated CRP in our patients with CD, as it has been reported in several [1, 4] but not all studies [11, 22]. In contrast, in our patients with UC, there was no association between VD levels and CRP as it has also been observed in most [11, 22] but again not all relevant studies [5, 16]. Only a few studies have evaluated the association of stool calprotectin with VD levels in IBD patients. We found an inverse correlation between fecal calprotectin and VD levels in CD, as it has been reported in other studies [10, 11, 16], but not in UC, which is in contrast to findings from other studies reporting such an inverse relationship in UC as well [10, 11, 16].

Data regarding VD levels in IBD patients and controls are conflicting [21, 25], with most studies being in line with our results, which may be due to a genetic explanation, as Greek people are often found to have low levels of VD [2]. Similarly, most studies observed no difference in VD levels between CD and UC patients [11, 13, 24], although few studies suggested lower VD levels in CD or in UC [3, 5].

Although only a small number of our patients had consecutive serum samples during anti-TNF therapy, a trend for increasing VD levels under treatment was observed. Similar findings have been reported in a study including CD patients receiving adalimumab [25], but not in two other studies including CD patients under anti-TNFa induction therapy [2] and IBD patients under one year of infliximab [26].

It is of note that as the exact role of VD in IBD has not yet been elucidated, although many studies have tried to examine this issue by evaluating 25(OH)D levels. It is under consideration whether assessment of other forms of VD, such as free 25(OH)D- or VD metabolites [eg its active form 1,25(OH)_2_D or 24,25(OH)_2_D] could provide greater insight into our knowledge. However, few efforts so far have been inconclusive [2, 12]. As limited data are available nowadays, the potential role of VD metabolites as biomarkers in IBD needs further investigation.

According to our knowledge, this is the first study that evaluated not only the levels of VD in Greek patients with IBD but also their associations with patient and disease characteristics including endoscopic and histological parameters. Moreover, this is one of the few studies evaluating the association of VD levels with stool calprotectin in CD or UC patients. On the other hand, there are some limitations. First, a relatively small number of patients was included, as strict exclusion criteria were applied in order to exclude conditions potentially affecting VD levels. Second, details on dietary habits were not available, although our patients supported adherence to Mediterranean diet, which has been shown to be associated with adequate VD intake [7]. In the same line, no questionnaire for physical activity and time of daily sun exposure were used.

In conclusion, low VD levels are detected in the majority of Greek IBD patients and are related to clinical activity. In CD, higher CRP and stool calprotectin are usually detected in patients with low than normal VD levels, rendering VD a possible activity marker, something that cannot be supported for UC patients. Given the controversial available information, large prospective studies are needed in order to clarify the role of VD levels in IBD patients.

## Data Availability

Data are available by the corresponding author upon reasonable request.
